# Risk for developing perianal abscess in type 1 and type 2 diabetes and the impact of poor glycemic control

**DOI:** 10.1007/s00384-020-03818-1

**Published:** 2020-12-17

**Authors:** Karin Adamo, Ulf Gunnarsson, Katarina Eeg-Olofsson, Karin Strigård, Fredrik Brännström

**Affiliations:** 1 Department of Surgical and Perioperative Science, Umeå University, 901 85 Umeå, Sweden; 2 Department of Molecular and Clinical Medicine, Sahlgrenska Academy, University of Gothenburg, Gothenburg, Sweden; 3 Department of Medicine, Sahlgrenska University Hospital, Gothenburg, Sweden; 4 Department of Surgery, Södertälje Hospital, Södertälje, Sweden

**Keywords:** Perianal abscess, Diabetes, HbA1c, Glycemic control

## Abstract

**Purpose:**

The primary aim of this study was to see whether perianal abscess rate differs between patients with type 1 and type 2 diabetes. A secondary aim was to determine whether poor glycemic control increases the risk for perianal abscess.

**Methods:**

Data from the Swedish National Diabetes Registry and the Swedish National Patient Registry between January 2008 and June 2015 were matched. The risk for anal abscess was evaluated in univariate and multivariate analyses with type of diabetes, HbA1c level, BMI, and various diabetes complications as independent factors.

**Results:**

Patients with type 1 diabetes had a lower rate of perianal abscess than patients with type 2 diabetes when adjusted for HbA1c, sex, and age (OR 0.65; 95% CI 0.57–0.73). The risk for perianal abscess increased with higher HbA1c. Incidence of perianal abscess was also elevated in diabetes patients with complications related to poor glycemic control such as ketoacidosis and coma (OR 2.63; 95% CI 2.06–3.35), gastroparesis, and polyneuropathy (OR 1.81; 95% CI 1.41–2.32).

**Conclusions:**

The prevalence of perianal abscess was higher among patients with type 2 diabetes than those with type 1, suggesting that metabolic derangement may be more important than autoimmune factors. Poor glycemic control was associated with higher risk for perianal abscess.

## Introduction

Perianal abscess is a common disease where surgical intervention is the only adequate treatment [1, 2]. Some individuals suffer only one episode, while others continue to have problems due to incomplete healing, recurrence, fistula, and/or unsuccessful drainage. The use of antibiotics after surgical drainage to avoid recurrence and complications such as perianal fistula is a matter of debate. Studies on this topic are sparse, and the degree of evidence is low [1–3]. One known risk factor for fistula formation is age below 40 years [4]. The incidence of perianal abscess is higher among men and younger individuals, and the mean age for debut of a perianal abscess is estimated to be around 43 years [5]. The incidence of surgically drained abscess in Sweden is 16 per 100,000 inhabitants per year [5]. Many risk factors have been considered, but more specific evidence-based information is required if we are to gain a better understanding of this disease and improve its treatment, thereby increasing the proportion of patients with uncomplicated recovery.

It has been established that diabetes is a risk factor for infections of the skin, urinary tract, and lower respiratory tract [6]. It has also been specifically associated with the occurrence of perianal abscess. Wei et al. concluded that the chance of being diagnosed with diabetes increases after an episode of perianal abscess [7]. In an investigation on the relationship between diabetes and occurrence, as well as recurrence, of perianal abscess, an association was found between diabetes and occurrence, but not recurrence [5]. However, this study did not distinguish between type 1 and type 2 diabetes.

Type 1 diabetes is caused by autoimmune destruction of β-cells. While this pathogenesis may play a role in type 2 diabetes, the overwhelming pathogenetic factors in type 2 diabetes are insulin resistance, β-cell dysfunction, and metabolic stress related to lifestyle and obesity. Patients who develop type 1 diabetes are usually young, and the duration of disease is lifelong, whereas patients with type 2 diabetes are older, and the duration of disease is thus shorter [8]. Hyperglycemia is the key feature in both type 1 and type 2 diabetes. Though the risk for perianal abscess is higher in diabetic patients, this association may differ between type 1 and type 2 diabetes due to the difference in pathogenesis and treatment regimens. As with many other diabetes complications, it is possible that the risk for perianal abscess is related to poor glycemic control with hyperglycemia, though this association is not clear [9].

Perianal abscess is more common in certain patient groups with diffuse perianal symptoms. If these groups can be defined, then patients with perianal symptoms should be investigated for perianal abscess. The reverse may also be the case, i.e., a patient with perianal abscess may belong to one of those groups. If patients with diabetes type 1 or type 2 and/or poorly regulated diabetes are at specific risk for perianal abscess, then a patient with perianal abscess should be investigated for diabetes. Identification of risk factors may also help us to further understand the pathogenesis of perianal abscess.

The primary aim of this study was to investigate whether increased risk for perianal abscess depends on diabetes and specifically the type of diabetes. Secondary aims were to investigate the roles of poor glycemic control and high BMI in the development of perianal abscess in patients with diabetes.

## Material and methods

### Registries

Data from the Swedish National Patient Registry (NPR) and the Swedish National Diabetes Registry (NDR) were matched to form a merged database. The NPR includes all diagnoses according to ICD 10 from hospital admissions throughout Sweden, and registration is regulated by law. All patients receiving outpatient treatment at a specialized clinic are also included. These registries do not cover diagnosis and interventions by primary care physicians, and surgical procedures in this setting are practically non-existent. The NPR registry has been reviewed and shown to have high validity and 99% completeness for most inpatient diagnoses [10, 11].

The Swedish NDR was started 1996 and is a nationwide quality registry covering diabetes patients 18 years and older. Patient data are updated at least once each year [12]. The number of patients included is constantly increasing, partly due to the increase in the prevalence of diabetes and partly to more efficient registration. Completeness of the NDR differs between regions in Sweden, but based on data from the NPR that 4% of the Swedish population has diabetes, completeness in 2016 was estimated to exceed 90% [12, 13]. Each Swedish citizen has a unique personal identity number that enables cross-matching of otherwise unrelated registries such as the NPR and the NDR [14].

### Methods

Data from the NDR registered between January 1, 2008, and June 30, 2015, on type of diabetes, treatment, HbA1c, height, weight, blood lipid levels, smoking, sex, and age were retrieved. Patients with diabetes other than type 1 or type 2 diabetes (e.g., after pancreatectomy) were excluded. Data extracted from the NDR were cross-matched with data from the NPR over the same period of time to obtain information on diagnosis of perianal abscess (K61.0) and surgery for perianal abscess (JHA 00) in diabetic patients. The definition of “perianal abscess” in the study required one of the codes for “perianal abscess” or “surgery for perianal abscess,” and the diagnosis was only used once for each person. Patients defined as having perianal abscess are thus those diagnosed with a perianal abscess at a hospital or specialized clinic (i.e., not a primary health center). Surgical treatment is the norm for treatment of perianal abscess at hospitals and specialized clinics in Sweden although rare exceptions treated by other means may also be included by this definition. Furthermore, the diagnoses ketoacidosis (E10.0A, E11.0A, E10.1A, E11.1A), hyperosmolar coma (E10.0B, E11.0B), gastroparesis (K31.8), and polyneuropathy (G61.9, E11.4D, E10.4D, G63.2) were extracted for analyses since they indicate poor glycemic control in diabetes.

Age was divided into four groups based on quartiles; 18–56, 57–66, 67–75, and 76 years and older. BMI was divided into six groups based on the WHO classification of obesity: (1) patients with BMI less than 25; (2) BMI 25–29; (3) BMI 30–34; (4) BMI 35–39; and (5) BMI 40 and above. Group 6 comprised patients with BMI missing. The National Board of Health and Welfare in Sweden has HbA1c < 52 mmol/mol as a treatment goal. In this study, patients were divided into six groups based on HbA1c level as follows: (1) HbA1c < 52 mmol/mol; (2) HbA1c 52–75 mmol/mol (75th percentile); (3) HbA1c 76–92 mmol/mol (90th percentile); (4) HbA1c 92–126 mmol/mol (99th percentile); (5) HbA1c 127 mmol/mol and above; and finally (6) patients whose HbA1c was missing. The value of HbA1c used was taken from the NDR and not necessarily sampled at the same time as the treatment of the perianal abscess.

Poor glycemic control in this study was defined as HbA1c > 52 mmol/mol at any point during the study period. Poor glycemic control is also a causal factor in the diagnoses associated with poorly controlled diabetes used in our analyses, namely, ketoacidosis, hyperosmolar coma, gastroparesis, and polyneuropathy.

Analyses involving ketoacidosis and hyperosmolar coma were made on type 1 diabetes patents only, since these rarely occur in type 2 except as a consequence of interference with other mechanisms.

### Statistical methods

Uni- and multivariate logistic regression analyses were used to compare differences in proportions. In the multivariate analyses, all variables were entered at the same time (force entry). Multicollinearity was checked for by analyzing the variance inflation factor (VIF). Differences in mean values were compared using the Mann-Whitney *U* test. All analyses were performed with STATA version 13.1 (StataCorp LP, College Station, TX, USA).

## Results

A total of 607 228 patients with diabetes were registered in the National Diabetes Registry (NDR) during the study period 2008–2015. After exclusion of cases with diabetes other than type 1 and type 2, 568,908 patients remained (58,454 with type 1 diabetes and 510,454 with type 2 diabetes). The perianal abscess rate for the entire cohort was 0.49% (2782/568,908).

### Diabetes type 1 and 2

In univariate analyses, patients with type 1 diabetes had a higher odds ratio for perianal abscess. When correcting for HbA1c, sex, and age in the multivariate analyses, however, this relationship was reversed with a lower odds ratio (OR) of 0.73 (0.64–0.83) for patients with diabetes type 1 for the occurrence of a perianal abscess compared to patients with diabetes type 2 (Table 1). When correcting for age only, the relationship was also reversed with an OR of 0.67 (0.59–0.75) for type 1 diabetes patients. This was not the case when correcting for HbA1c (OR 1.02 (0.91–1.15) for type 1 diabetes patients), sex (OR 1.18 (1.06–1.33) for type 1 diabetes patients), or BMI (OR 1.40 (1.24–1.57) for type 1 diabetes patients).

**Table 1:** Type of diabetes and level of HbA1c and BMI on the risk for perianal abscess

			Univariate			Multivariate		
	Abscess (*n*)	Risk	OR	95% CI	*p* Value	OR	95% CI	*p* Value
Diabetes 1	331/58 454	0.57%	1.18	1.05–1.32	0.005	0.73	0.64–0.83	< 0.001
Diabetes 2	2451/510,454	0.48%	1.00					
BMI < 25	393/105,193	0.37%	1.00					
BMI 25–30	920/208,144	0.40%	1.18	1.05–1.33	0.005	1.08	0.96–1.22	0.207
BMI 30–35	782/138,423	0.56%	1.51	1.34–1.71	< 0.001	1.25	1.10–1.42	0.001
BMI 35–40	347/53,097	0.65%	1.71	1.52–2.03	< 0.001	1.32	1.13–1.53	< 0.001
BMI > 40	200/23,374	0.86%	1.98	1.94–2.73	< 0.001	1.61	1.35–1.93	< 0.001
BMI unknown	140/40,677	0.35%	2.65	0.76–1.12	0.404	1.01	0.83–1.24	0.913
HbA1c > 52	559/156,794	0.35%	1.00					
HbA1c 52–75	1069/260,621	0.41%	1.15	1.03–1.28	0.007	1.10	0.99–1.22	0.074
HbA1c 76–92	538/85,801	0.63%	1.76	1.57–1.99	< 0.001	1.47	1.30–1.66	< 0.001
HbA1c 93–127	496/53,087	0.93%	2.61	2.31–2.95	< 0.001	1.93	1.70–2.19	< 0.001
HbA1c > 127	93/5558	1.67%	4.68	3.75–5.83	< 0.001	3.38	2.70–4.22	< 0.001
HbA1c unknown	27/6458	0.42%	1.17	0.80–1.73	0.418	1.30	0.87–1.94	0.196
Female	782/246,769	0.32%	0.51	0.47–0.55	< 0.001	0.56	0.51–0.61	< 0.001
Male	2000/322,139	0.63%	1.00					
Age 18–56	1316/135,537	0.97%	5.41	4.73–6.18	< 0.001	4.55	3.96–5.24	< 0.001
Age 57–66	716/141,922	0.50%	2.80	2.43–3.23	< 0.001	2.35	2.04–2.72	< 0.001
Age 67–75	492/148,810	0.33%	1.83	1.57–2.12	< 0.001	1.65	1.42–1.92	< 0.001
Age > 76	258/142,639	0.18%	1.00					

Uni- and multivariate logistic regression analyzing the impact of type of diabetes and the level of HbA1c and BMI on the risk for perianal abscess. *n* total number of patients, 95% CI 95% confidence interval. Mean VIF 1.59

### Age and diabetes

**Fig. 1:**
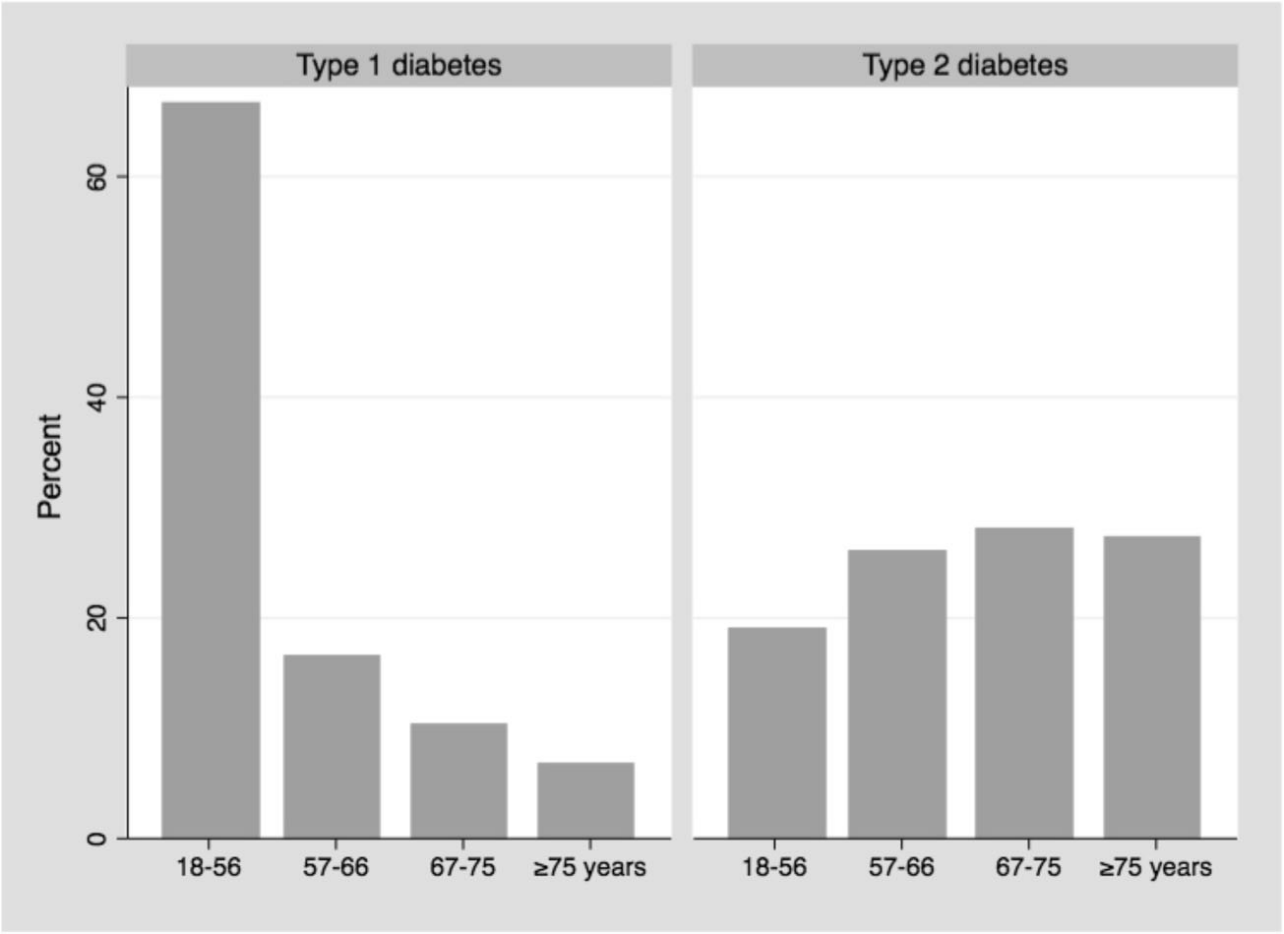
Age distribution of type 1 and type 2 diabetes in the NDR 2008-2015, type 1 diabetes (n=58454) and type 2 diabetes (n=510454)

**Fig. 2:**
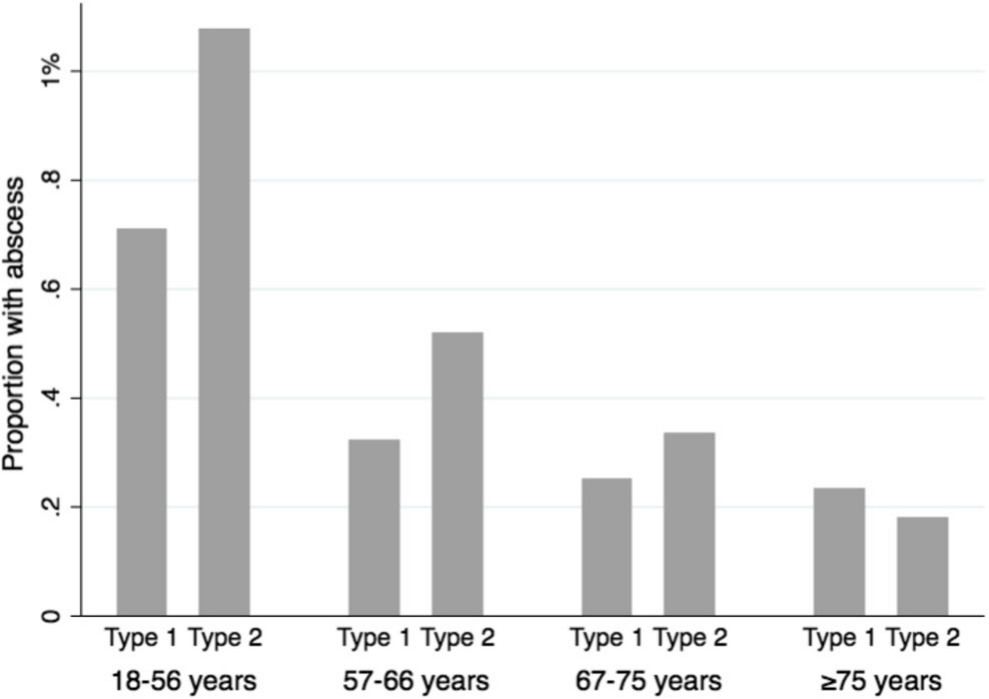
Proportion of patients with perianal abscess in each age group

Age distribution differed between type 1 diabetes and type 2 (Fig. 1). Median age of type 1 diabetes patients was 47 years and 68 years for type 2 (*p* value < 0.001). The proportion of patients with perianal abscess with type 1 and type 2 diabetes in each age group was compared (Fig. 2). In the younger age groups, type 1 diabetes patients had lower OR for perianal abscess. For age group 18–56 years, the OR was 0.66 (0.57–0.75) with a *p* value of < 0.001; for age group 57–66, the OR was 0.62 (0.43–0.89) with a *p* value of 0.009; for age group 67–75, the OR was 0.75 (0.45–1.26) with a *p* value of 0.275; and for age group > 75, the OR was 1.29 (0.43–0.89) with a *p* value of 0.453.

### Impact of HbA1c levels and BMI

The HbA1c level 52–75 mmol/mol group had an increased OR (1.10 (0.99–1.22)) for perianal abscess compared to the group below 52 mmol/mol adjusted for age and sex. OR increased with increasing HbA1c level (Table 1). Patients with diabetes and BMI > 25 had a higher risk for perianal abscess compared to patients with diabetes and BMI < 25, in both the uni- and multivariate models, the OR increasing with increasing BMI (Table 1).

### Effects of complications to diabetes

Type 1 diabetes patients with a diagnosis of ketoacidosis and/or hyperosmolar coma had more than double the OR (2.31 (1.72–3.12)) of having a perianal abscess even when adjusting for age and sex (Table 2). Diabetes patients with a diagnosis of gastroparesis and/or polyneuropathy also had increased OR (1.81 (1.41–2.32)) (Table 3) for having av perianal abscess. Women had a lower risk for developing a perianal abscess in all analyses. Persons older than 76 years also had a lower risk for developing an abscess in all analyses, the OR increasing with decreasing age (Tables 1, 2, and 3).

**Table 2:** Ketoacidosis/coma on the risk for perianal abscess in patients with diabetes type 1

			Univariate			Multivariate		
	Abscess (*n*)	Risk	OR	95% CI	*p* Value	OR	95% CI	*p* Value
Ketoacidosis	52/4104	1.27%	2.49	1.85–3.35	< 0.001	2.31	1.72–3.12	< 0.001
No ketoacidosis	279/54,350	0.51%						
Female	118/25,665	0.46%	0.71	0.56–0.89	0.003	0.71	0.57–0.89	0.003
Male	213/32,789	0.65%	1.00					
Age 18–56	276/38,928	0.71%	3.07	1.58–5.98	< 0.001	2.82	1.45–5.49	0.002
Age 57–66	31/9660	0.32%	1.39	0.66–2.92	0.387	1.33	0.63–2.80	0.453
Age 67–75	15/5977	0.33%	1.08	0.47–2.48	0.847	1.05	0.46–2.40	0.907
Age > 76	9/3889	0.18%	1.00					

Uni- and multivariate logistic regression analyzing the impact of ketoacidosis/coma on the risk for perianal abscess in patients with diabetes type 1. *n* total number of patients, *95% CI* 95% confidence interval. *Ketoacidosis* a diagnosis of ketoacidosis and/or hyperosmolar coma during the study period. Mean VIF 1.29

**Table 3:** Gastroparesis and polyneuropathy on the risk for perianal abscess

			Univariate			Multivariate		
	Abscess (*n*)	Risk	OR	95% CI	*p* Value	OR	95% CI	*p* Value
Type 1 diabetes	331/58,454	0.57%	1.18	1.05–1.32	0.005	0.67	0.59–0.75	< 0.001
Type 2 diabetes	2451/510,454	0.48%	1.00			1.00		
Neuropathy	67/7932	0.85%	1.75	1.37–2.23	< 0.001	1.81	1.41–2.32	< 0.001
No neuropathy	2748/566,686	0.48%	1.00			1.00		
Female	664/221,104	0.30%	0.51	0.47–0.55	< 0.001	0.56	0.51–0.61	< 0.001
Male	1787/289,350	0.51%	1.00			1.00		
Age 18–56	1316/135,537	0.97%	5.41	4.73–6.18	< 0.001	0.47	0.43–0.52	< 0.001
Age 57–66	716/141,922	0.50%	2.80	2.43–3.23	< 0.001	0.31	0.28–0.35	< 0.001
Age 67–75	492/148,810	0.33%	1.83	1.57–2.12	< 0.001	0.18	0.16–0.21	< 0.001
Age ≥ 76	258/142,639	0.18%	1.00			1.00		

Uni- and multivariate logistic regression analyzing the impact of gastroparesis and polyneuropathy on the risk for perianal abscess. *Neuropathy* diagnosis of gastroparesis and/or polyneuropathy, *n* total number of patients, 95% CI 95% confidence interval, *gastroparesis/polyneuropathy* a diagnosis of gastroparesis and/or polyneuropathy during the study period. Mean VIF 1.22

## Discussion

### Risk for perianal abscess and type of diabetes

In the multivariate analyses, patients with type 2 diabetes had a higher risk for developing a perianal abscess than patients with type 1 diabetes when adjusted for sex, age, and HbA1c level in this large population-based Swedish registry study. It is possible that symptoms of the metabolic syndrome such as increased blood pressure, excess body fat around the waist, and abnormal triglyceride levels cause stress that may play a part in mechanisms leading to the development of perianal abscess, and not necessarily the diabetes itself.

In contrast to the results of the multivariate analyses, type 1 but not type 2 diabetes patients were at risk for perianal abscess in the univariate analyses. This contrast is rare in statistics, but in this case, there is a logical explanation, the key variable being age. Further analyses of the different age groups revealed that type 2 diabetes was associated with a significantly greater risk for perianal abscess than type 1 diabetes in the younger age groups (18–56 and 57–66 years old, but not so in the older age groups 67–75 and ≥ 75 years). The mean age of patients with type 1 diabetes was much lower than for those with type 2 diabetes. As a result, the overall proportion of patients with a perianal abscess was higher in the type 1 diabetes group due to their lower age.

### The impact of poor glycemic control

The proportional relationship between HbA1c level and the risk for perianal abscess when corrected for age, sex, type of diabetes and BMI indicated that poor glycemic control is an important factor. Severe diabetic complications, i.e., hyperosmolar coma, ketoacidosis, gastroparesis, and polyneuropathy, all emanate from inadequately treated diabetes with poor glycemic control and are also significant risk factors for the development of perianal abscess. This stresses the importance of poor glycemic control in the predisposition of patients with diabetes to develop perianal abscess.

Several studies have shown that diabetes with poor glycemic control is a risk factor for severe skin infections [6, 15, 16]. Possible mechanisms include impaired neutrophil granulocyte function, impairment of the antioxidant system, and deterioration in organ function secondary to vascular complications arising from diabetes [6, 15, 16]. Such skin infections may lead to the diagnosis of diabetes but can also occur after diabetes has been diagnosed. Other explanations include oxidative stress and nutritional deficiency. Adipose tissue monitors the extracellular environment and takes an active part in the secretion of inflammatory mediators. This may play an important role [16, 17]. Perianal abscess may be classified as a skin infection, though it is usually defined as an infection of the cryptoglandular glands close to the anus.

In a previous study, a relationship was described between diabetes and the occurrence, but not recurrence, of perianal abscess [5]. A reasonable interpretation of the results of the present study is that a patient with poor glycemic control runs an increased risk for perianal abscess, and that if glycemic control (measured as HbA1c) improves, the risk for recurrence decreases.

### The impact of obesity

High BMI was shown to be an independent risk factor for perianal abscess in patients with diabetes. This relationship has not been described before although recurrence of perianal abscess has been associated with obesity [5]. Several previous studies have also revealed an association between obesity and wound healing [17, 18]. Obesity has a negative impact on the function of adipocytes and on the immune surveillance system. This is thought to lead to deregulation of the immune response predisposing to such conditions as skin infection, pancreatitis, and pulmonary infection [19]. Another theory is that activation of T-cell metabolism is disturbed in obese patients, interfering with the immune response to acute and chronic infections [20].

Cottam et al investigated the importance of inflammatory mediators such as hormones and cytokines in obesity [17]. Elevated levels of angiotensinogen, transforming growth factor beta, tumor necrosis factor alpha, and interleukin 6 were observed in obese patients. All these markers have been associated with inflammatory conditions and sepsis [17, 19] and may further increase the risk for perianal abscess in diabetic patients with obesity. Green et al. also discussed how excess of nutrients and adipocytes may activate T-cells in a pathogen-free environment [20]. Though not all mechanisms are clear, it may be concluded that T-cells seem to play an important role in the relationship between obesity and infection.

### Weaknesses and strength of this study

All registry-based epidemiologic studies have shortcomings associated with this method. Firstly, a registry contains the data included at registration, and it is not possible to extend the variables included in order to refine any analysis that provides an interesting result. Secondly, different variables may be registered with differing degrees of precision. In the case of the NDR, there may still be an element of selective fall-off, despite the high level of completeness. Thirdly, the high number of patients included may give rise to significant differences that lack clinical relevance, and the specific clinical role of each result must be evaluated.

The study data constitute of ICD codes registered between 2008 and 2015. Unfortunately, it is not possible to determine the chronologic relationship between a high HbA1c level or a specific diagnosis and an episode of perianal abscess. Thus, it is not possible to state with certainty which of perianal abscess or poor glycemic control is the risk factor or the outcome. However, such a reverse in relationship seems unlikely for the risk factors diabetes/type of diabetes and BMI.

It is possible that small abscesses were drained by a small incision or managed by some other method within the primary care system or by the patients themselves. However, the proportion of manifest perianal abscesses treated by incision outside a hospital is small, though we do not have available data to support this.

Another weakness in the study is that data were missing for some variables. However, the numbers were low, and the missing data category for each variable did not differ significantly from the reference category in any analysis indicating that missing points were random.

The strength of the present study is that it is based on a large population-based cohort with data gathered from two well-established registries with high validity and a high degree of completeness. The generalizability of the results may thus be considered high, mirroring the impact of those variables on the entire population treated in the various healthcare sectors. This study thus contributes new information regarding a common disease, i.e., perianal abscess in a specific group of patients at risk, i.e., diabetes mellitus.

Another strength is that both the effects of a modest deviation from good glycemic control (HbA1c 52–75) and a very poor glycemic control (HbA1c > 127 and diagnosis associated with very poor glycemic control) are analyzed in the same study. Our conclusions are strengthened by the fact that more obvious effects were seen in patients presenting with very poor glycemic control as indicated by the variables HbA1c > 127, ketoacidosis, and other complications to diabetes.

## Conclusion

The prevalence of perianal abscess among patients with type 2 diabetes was higher than that seen in type 1 diabetes when comparing patients within specific age groups. This suggests that factors other than autoimmune disease predispose to this condition and that metabolic factors play an important role. Poor glycemic control clearly predisposed to the development of perianal abscess. High BMI may also be a risk factor for perianal abscess, and this association will be the subject of future studies by this group.

## Data Availability

Databases are available upon request from the appropriate party.
